# How GRAIL controls Treg function to maintain self-tolerance

**DOI:** 10.3389/fimmu.2022.1046631

**Published:** 2022-12-08

**Authors:** C. Garrison Fathman, Linda Yip, Diana Gómez-Martín, Mang Yu, Christine M. Seroogy, Clarence R. Hurt, Jack T. Lin, Jennifer A. Jenks, Kari C. Nadeau, Luis Soares

**Affiliations:** ^1^ Department of Medicine, School of Medicine, Stanford University, Palo Alto, CA, United States; ^2^ Departamento de Inmunología y Reumatología, Instituto Nacional de Ciencias Médicas y Nutrición Salvador Zubirán (INCMNSZ), Mexico City, Mexico; ^3^ Department of Pediatrics, School of Medicine, Stanford University, Palo Alto, CA, United States; ^4^ Department of Pediatrics, Division of Allergy, Immunology and Rheumatology, University of Wisconsin, Madison, WI, United States; ^5^ IL-2Rx, San Jose, CA, United States; ^6^ Sean N. Parker Center for Allergy & Asthma Research, School of Medicine, Stanford University, Palo Alto, CA, United States

**Keywords:** GRAIL, regulatory T cell, neddylation, cullin RING ligase, immune regulation, low dose IL-2, protein drug conjugates

## Abstract

Regulatory T cells (T_regs_) normally maintain self-tolerance. T_regs_ recognize “self” such that when they are not working properly, such as in autoimmunity, the immune system can attack and destroy one’s own tissues. Current therapies for autoimmunity rely on relatively ineffective and too often toxic therapies to “treat” the destructive inflammation. Restoring defective endogenous immune regulation (self-tolerance) would represent a paradigm shift in the therapy of these diseases. One recent approach to restore self-tolerance is to use “low dose IL-2” as a therapy to increase the number of circulating T_regs_. However, studies to-date have not demonstrated that low-dose IL-2 therapy can restore concomitant T_reg_ function, and phase 2 studies in low dose IL-2 treated patients with autoimmune diseases have failed to demonstrate significant clinical benefit. We hypothesize that the defect in self-tolerance seen in autoimmunity is not due to an insufficient number of available T_regs_, but rather, due to defects in second messengers downstream of the IL-2R that normally control T_reg_ function and stability. Previous studies from our lab and others have demonstrated that GRAIL (a ubiquitin E3 ligase) is important in T_reg_ function. GRAIL expression is markedly diminished in T_regs_ from patients with autoimmune diseases and allergic asthma and is also diminished in T_regs_ of mice that are considered autoimmune prone. In the relevant pathway in T_regs_, GRAIL normally blocks cullin ring ligase activity, which inhibits IL-2R desensitization in T_regs_ and consequently promotes T_reg_ function. As a result of this defect in GRAIL expression, the T_regs_ of patients with autoimmune diseases and allergic asthma degrade IL-2R-associated pJAK1 following activation with low dose IL-2, and thus cannot maintain pSTAT5 expression. pSTAT5 controls the transcription of genes required for T_reg_ function. Additionally, the GRAIL-mediated defect may also allow the degradation of the mTOR inhibitor, DEP domain-containing mTOR interacting protein (Deptor). This can lead to IL-2R activation of mTOR and loss of T_reg_ stability in autoimmune patients. Using a monoclonal antibody to the remnant di-glycine tag on ubiquitinated proteins after trypsin digestion, we identified a protein that was ubiquitinated by GRAIL that is important in T_reg_ function, cullin5. Our data demonstrate that GRAIL acts a negative regulator of IL-2R desensitization by ubiquitinating a lysine on cullin5 that must be neddylated to allow cullin5 cullin ring ligase activity. We hypothesize that a neddylation inhibitor in combination with low dose IL-2 activation could be used to substitute for GRAIL and restore T_reg_ function and stability in the T_regs_ of autoimmune and allergic asthma patients. However, the neddylation activating enzyme inhibitors (NAEi) are toxic when given systemically. By generating a protein drug conjugate (PDC) consisting of a NAEi bound, *via* cleavable linkers, to a fusion protein of murine IL-2 (to target the drug to T_regs_), we were able to use 1000-fold less of the neddylation inhibitor drug than the amount required for therapeutically effective systemic delivery. The PDC was effective in blocking the onset or the progression of disease in several mouse models of autoimmunity (type 1 diabetes, systemic lupus erythematosus, and multiple sclerosis) and a mouse model of allergic asthma in the absence of detectable toxicity. This PDC strategy represents targeted drug delivery at its best where the defect causing the disease was identified, a drug was designed and developed to correct the defect, and the drug was targeted and delivered only to cells that needed it, maximizing safety and efficacy.

## Introduction

**Figure 1:**
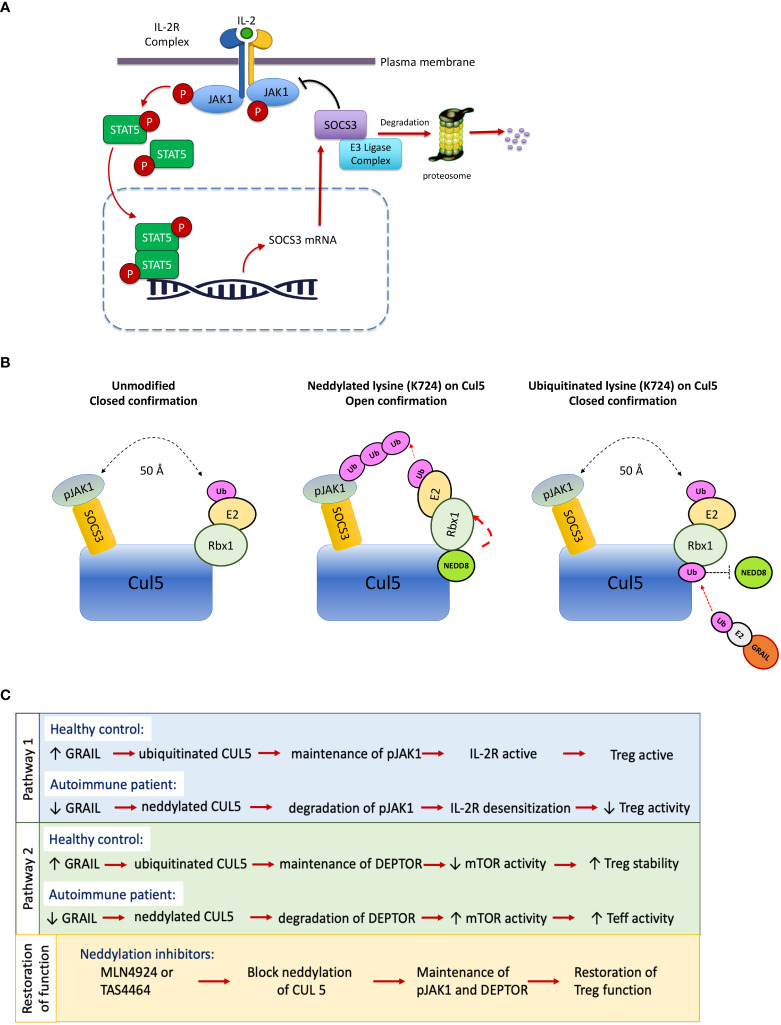
IL-2 receptor desensitization, neddylation of Cullin5 and restoration of T_reg_ function. Desensitization of the IL-2R means turning it off. SOCS3 is a negative regulator of IL-2Rβ chain–associated pJAK1 and forms a cullin ring ligase to ubiquitinate pJAK1. **(A)** Healthy individuals inhibit IL-2R desensitization in their T_regs_ by a protein, GRAIL, that is constitutively expressed in normal T_regs_. Patients with autoimmunity have a defect in this pathway of inhibition of desensitization. **(B)** Upon neddylation of Cullin5 (Cul5) at lysine 724, one end of the Rbx1 protein is untethered and undergoes a 50A shift, bringing the ubiquitin transferase (E2) in proximity of the target of the SOCS3, pJAK1, to transfer ubiquitin for degradation. GRAIL, constitutively expressed in T_regs_, is a competitive inhibitor of this process, as it ubiquitinates lysine 724 and does not allow neddylation and release of the E2 bound to the Rbx1 protein, thus inhibiting IL-2R desensitization. **(C)** Reduced GRAIL expression in autoimmune patients leads to diminished T_reg_ function *via* two distinct pathways that favor the neddylation of CUL5. Treatment with neddylation inhibitors such as MLN9424 may restore T_reg_ activity.

Autoimmune diseases can develop due to a defect in peripheral regulatory T cells (T_regs_) (1–4). There have been several anecdotal reports of the successful use of low dose interleukin 2 (IL-2) to treat autoimmune diseases (5, 6). However, as recently reported, placebo-controlled phase 2 clinical trials using low dose IL-2, muteins of IL-2, or pegylated IL-2 as a potential therapy to treat autoimmune diseases have not reported statistically significant clinical responses, despite a significant increase in the level of circulating T_regs_ in the treated patients (5). Although there are data showing that the defect in Tregs in autoimmunity and allergy may be due to insufficient numbers of Tregs, here we present data to suggest that a defect in IL-2 receptor (IL-2R) signaling in Tregs leads to diminished Treg function and underlies both autoimmune diseases and allergic asthma. In this study, we compared the responses to low dose IL-2 *in vitro*, of equal numbers of Tregs isolated from patients with autoimmune diseases or allergic asthma to Tregs from healthy controls, to ask if the defect might be in the number of Tregs. Rather than the defect being in the number of Tregs (as studied *in vitro*) our studies identified a common druggable defect in IL-2 receptor second messenger signaling that was shared among patients with autoimmune diseases and patients with severe food allergy. We found that T_regs_ from these patients lost inhibition of IL-2R desensitization (**Figure 1A**), which equates to a loss of regulatory function. When activated with low dose IL-2 *in vitro*, T_regs_ from healthy subjects inhibit IL-2R desensitization to prolong pSTAT5 and Deptor expression for ~4 hours, allowing the genes for T_reg_ function to be transcribed *via* pSTAT5 to maintain the T_reg_ phenotype (7), and inhibiting mTOR activation to maintain T_reg_ stability *via* Deptor (8, 9). Deptor is a mTOR inhibitor and functions similarly to the immunosuppressant Rapamycin, although by a distinct mechanism of action (9). In patients with autoimmune diseases or allergic asthma, there is a defect in the inhibition of IL-2R desensitization. Following activation with low dose IL-2, cullin ring ligase (CRL) degradation of IL-2Rβ chain-associated pJAK1 and Deptor occur, leading to rapid reduction in both pSTAT5 and Deptor expression. This diminishes transcription of the genes required for regulation and allows activation of mTOR that leads to a loss of T_reg_ stability.

T_regs_ from healthy controls constitutively express a ubiquitin E3 ligase called GRAIL (Gene Related to Anergy In Lymphocytes) (10) that we found to ubiquitinate the exact lysine (K724) on Cul5 proteins that needs to be neddylated as a condition for CRL activation. Once neddylated, the ubiquitin transferase (E2) attached to the Rbx1 protein on the cullin5 backbone undergoes a 50A shift to bring the E2 in proximity of the target of the CRL to allow transfer of ubiquitin. The SOCS3/Cullin5 CRL ubiquitinates pJAK1 (9, 10). Deptor is also degraded by an activated cullin5 CRL, the F box protein βTrCP of the Cul5/Elongin B complex (11). Following IL-2R activation, poly-ubiquitination by the Cullin 5 CRLs leads to proteasomal degradation of pJAK1 and Deptor, desensitizing (turning off) the IL-2R signaling (**Figure 1B**). In T_regs_ from healthy controls, GRAIL acts as a competitive inhibitor of neddylation by ubiquitinating the exact lysine on cullin5 that needs to be neddylated to activate the CRL. Thus, in Tregs, GRAIL acts as a competitive inhibitor of neddylation and blocks activation of the cullin5 CRLs that normally degrade pJAK1 and Deptor in IL-2 activated CD4 T effector cells (11–13). GRAIL protein but not *GRAIL* mRNA expression is reduced in T_regs_ from SLE patients. The competitive inhibition of neddylation by GRAIL in the T_regs_ from the patients with SLE is thus diminished with a resultant loss of inhibition of IL-2R desensitization and prolongation of IL-2R signaling (**Figure 1C**). These defects can be corrected by the application of a small molecule drug called a neddylation activating enzyme inhibitor (NAEi) that replaces the function of GRAIL to block neddylation and inactivate the cullin5 ring ligases (14).

Our studies propose a paradigm shift in immune therapy away from immunosuppression to restoration of self-tolerance. These studies demonstrate that repairing the defect in T_reg_ function can restore normal inhibition of desensitization of the T_reg_ IL-2R to low dose IL-2 and allow transcription of the genes required for regulatory function and block the activation of mTOR to maintain the T_reg_ phenotype (**Figure 1C**). As described below, we developed a novel protein drug conjugate (PDC) of a fusion protein of murine IL-2 and thioredoxin to which we attached three molecules of a neddylation activating enzyme inhibitor (MLN4924) (14) by cleavable linkers (15). The PDC blocks the neddylation dependent activation step of the cullin5 ring ligases that controls pJAK1 and Deptor degradation. As shown below, the PDCs were effective in blocking progression of disease in animal models of autoimmunity and asthma.

## Identification of a defect in inhibition of T_reg_ IL-2R desensitization in autoimmunity

**Figure 2:**
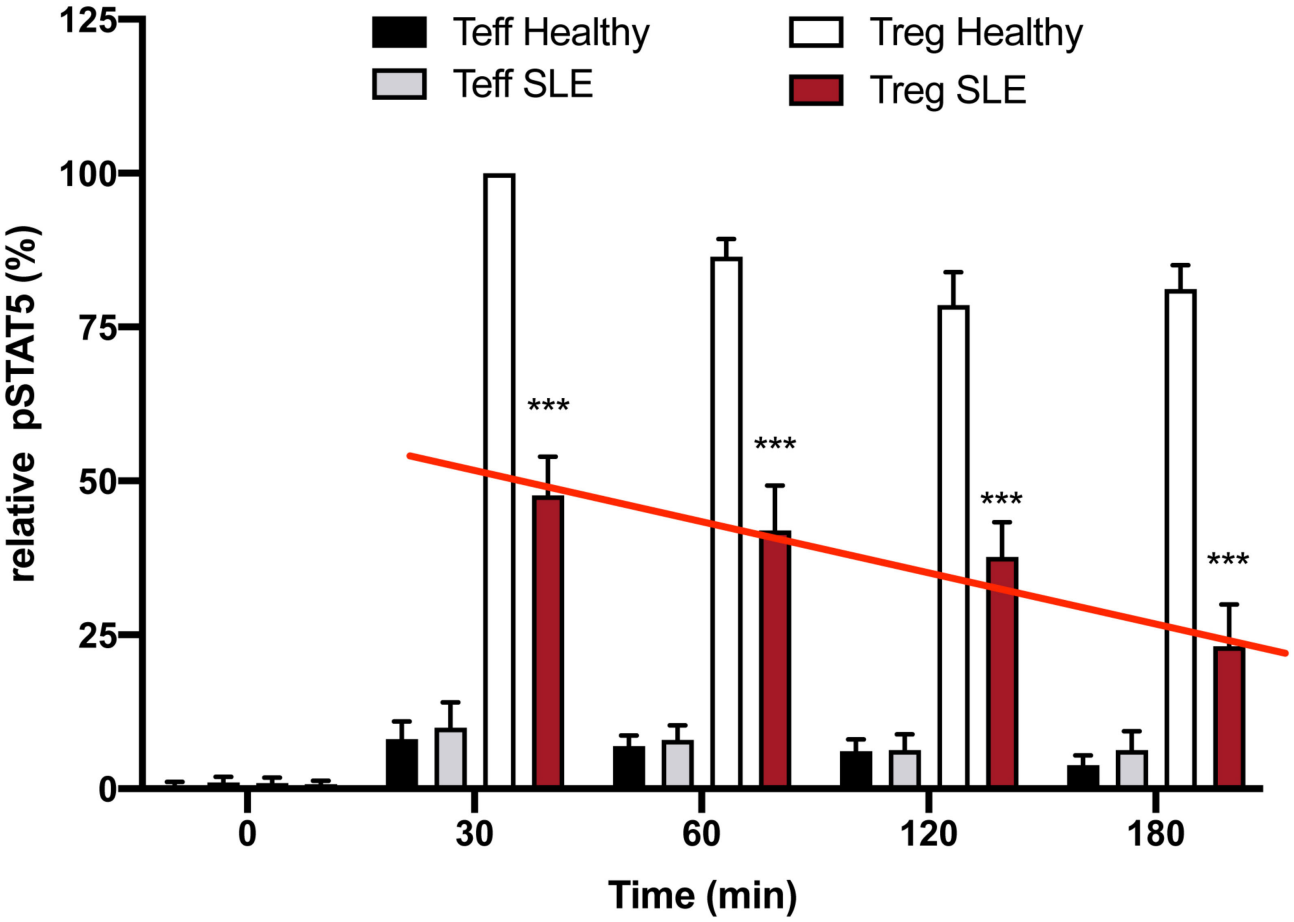
Defective inhibition of IL-2 desensitization in the T_regs_ of SLE patients. The 25 SLE patients studied were selected from a clinic in Mexico City irrespective of current disease status. These graphs represent Western blot data of pSTAT5 phosphorylation in T_effs_ and T_regs_ of the SLE patients and healthy sex and age-matched controls after stimulation with low dose IL-2 (1 ng/ml; 25 IU/ml) for the indicated amounts of time. Expression is shown as a percent of pSTAT5 measured from 30 min-stimulated control T_regs_. A defect in the inhibition of IL-2R desensitization is observed in the T_regs_ of SLE patients (red line) n=6 per group ***p < 0.001 by 2-way ANOVA.

We hypothesize that restoration of peripheral tolerance in T_regs_ of autoimmune/allergy patients requires enhancement of T_reg_ function (restoration of downstream messenger effects) rather than simply an increase in the number of poorly functioning circulating T_regs_. To address this hypothesis, we asked if the same number of T_regs_ isolated from patients with systemic lupus erythematosus (SLE, one of the diseases that has been treated with low dose IL-2 (16), would have a similar, or less robust response to low dose IL-2 *in vitro* when compared to T_regs_ from healthy controls. If their response to IL-2 activation was lower despite using the same number of Tregs from the SLE patients, this would suggest that there was a defect in their function. Dr. Diana Gomez-Martin, a former post-doc in the Fathman lab, isolated and then studied T_regs_ from patients with SLE seen in her rheumatology clinic in Mexico City. Interestingly, equal numbers of T_regs_ from the SLE patients studied, regardless of disease activity status, expressed similar amounts of cell surface CD25 as the controls when measured by FACS (data not shown) but had less pSTAT5 30 minutes following low dose IL-2 activation *in vitro* and had a more rapid loss of pSTAT5 expression than that seen in T_regs_ from healthy controls when stimulated with low dose IL-2 (**Figure 2**). This diminished pSTAT5 expression represents a defect in the normal inhibition of IL-2R desensitization seen in T_regs_ from healthy controls that maintain pSTAT5 expression for ≥4 hours. We hypothesize that the lower initial expression and subsequent loss of pSTAT5 expression in the SLE Tregs leads to ineffective transcription of the genes required for Treg function.

## The role of ubiquitin ligases in autoimmunity

As described by Amy Lin and Tak Mak in 2007, ubiquitin E3 ligases play an important role in controlling immune regulation (17). Ubiquitin E3 ligases are important in maintenance of self-tolerance and in the suppression of autoreactive T cells (18, 19). These authors suggested the possibility that there could be ways to exploit the therapeutic potential of manipulating ubiquitination, particularly for autoimmune disorders. Although traditionally, the addition of a ubiquitin chain targets a protein for degradation by the proteasome, mono- or pauci-ubiquitination of the protein can specify a nonproteolytic fate. The studies described below support their suggestion.

## Could defective desensitization of the T_reg_ IL-2R be related to GRAIL expression?

The IL-2R is sensitized (turned on) by ligand engagement to activate the JAK1/STAT5 pathway. Prolonged IL-2R signaling in activated CD25+ CD4 T effector cells (T_eff_) could result in proliferation and potentially chronic inflammation. However, when the IL-2R on CD4 T_regs_ is activated, pSTAT5 dimerizes and translocates into the nucleus where it initiates the transcription of multiple genes, including negative regulators such as SOCS3. In turn, SOCS3 feeds back into the signaling cascade desensitizing the IL-2R by inactivating pJAK1 (20) (**Figure 1A**). SOCS3 desensitizes (turns off) IL-2R signaling, thereby creating a negative feedback loop (21). SOCS3 is a member of a family of SOCS proteins. This multi-member family of proteins is important in creating non-redundant feedback inhibition of tyrosine kinase cytokine receptor activation (22). The SOCS family consists of a group of eight intracellular proteins: SOCS 1–7 and CIS, all possessing an SH2 domain, C-terminal SOCS box, N-terminal extended SH2 subdomain, and a variable N-terminal region (23). The SOCS box can recruit factors to form an E3 ligase complex (a cullin ring ligase, CRL) that ubiquitinates the target protein, leading to its proteasomal degradation. The IL-2β chain–associated pJAK1 is degraded by the SOCS3/Cul5 CRL. T_regs_ from patients with SLE had a defect in inhibition of IL-2R desensitization, pSTAT5 levels were initially lower in the T_regs_ from the patients, and STAT5 phosphorylation in response to low-dose IL-2 and was lost more rapidly in the T_regs_ from the disease patients compared to the T_regs_ from healthy controls (**Figure 2**).

**Figure 3:**
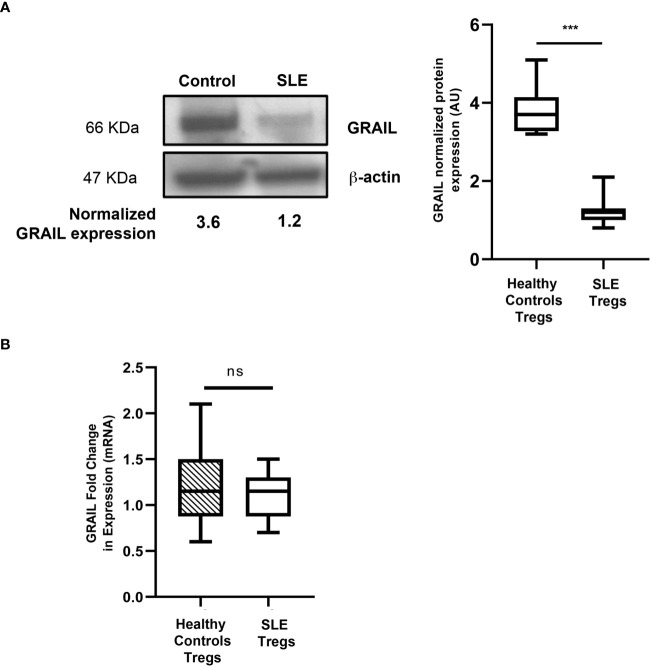
GRAIL expression in the Tregs of patients with SLE. **(A)** Immunoblotting data showing loss of GRAIL expression in the T_regs_ of a representative SLE patient vs. healthy control; the graph shows the pooled data in which SLE T_regs_ are deficient in GRAIL protein expression vs. healthy controls (HC=10, SLE=25 ***p < 0.0001 by unpaired T test). The T_regs_ from SLE patients were found to be deficient in GRAIL expression and had diminished T_reg_ function irrespective of disease activity (data not shown). **(B)** No differences were found in GRAIL mRNA levels in T_regs_ from SLE patients compared to healthy controls (HC=10, SLE=10 p=0.4715 by unpaired T-test). ns, not significant.

Studies from the Seroogy lab demonstrated that GRAIL, a single-protein ubiquitin ligase (8), was constitutively expressed in T_regs_ (24). These studies revealed that both GRAIL mRNA and protein expression were increased in naturally occurring thymically derived T_regs_ (mRNA levels were 10-fold higher compared to CD25- T cells). These studies also demonstrated that CD25+ Foxp3+ antigen- specific T cells were induced after a “tolerizing-administration” of antigen, and that GRAIL mRNA expression was upregulated in the CD25+ Foxp3+ antigen-specific subset. Collectively, these data demonstrated that GRAIL was differentially expressed in naturally occurring and peripherally induced CD25+ T_regs_ and that the expression of GRAIL was linked to their functional regulatory activity. By studying GRAIL expression in the T_regs_ isolated from patients with SLE, Dr. Gomez-Martin demonstrated a statistically significant reduction in the amount of GRAIL protein expressed in the T_regs_ from the SLE patients compared to the healthy controls, regardless of disease activity (**Figure 3**). Diminished GRAIL expression was also seen in T_regs_ from mice that are autoimmune prone (25).

## How could GRAIL expression influence IL-2R desensitization?

**Figure 4:**
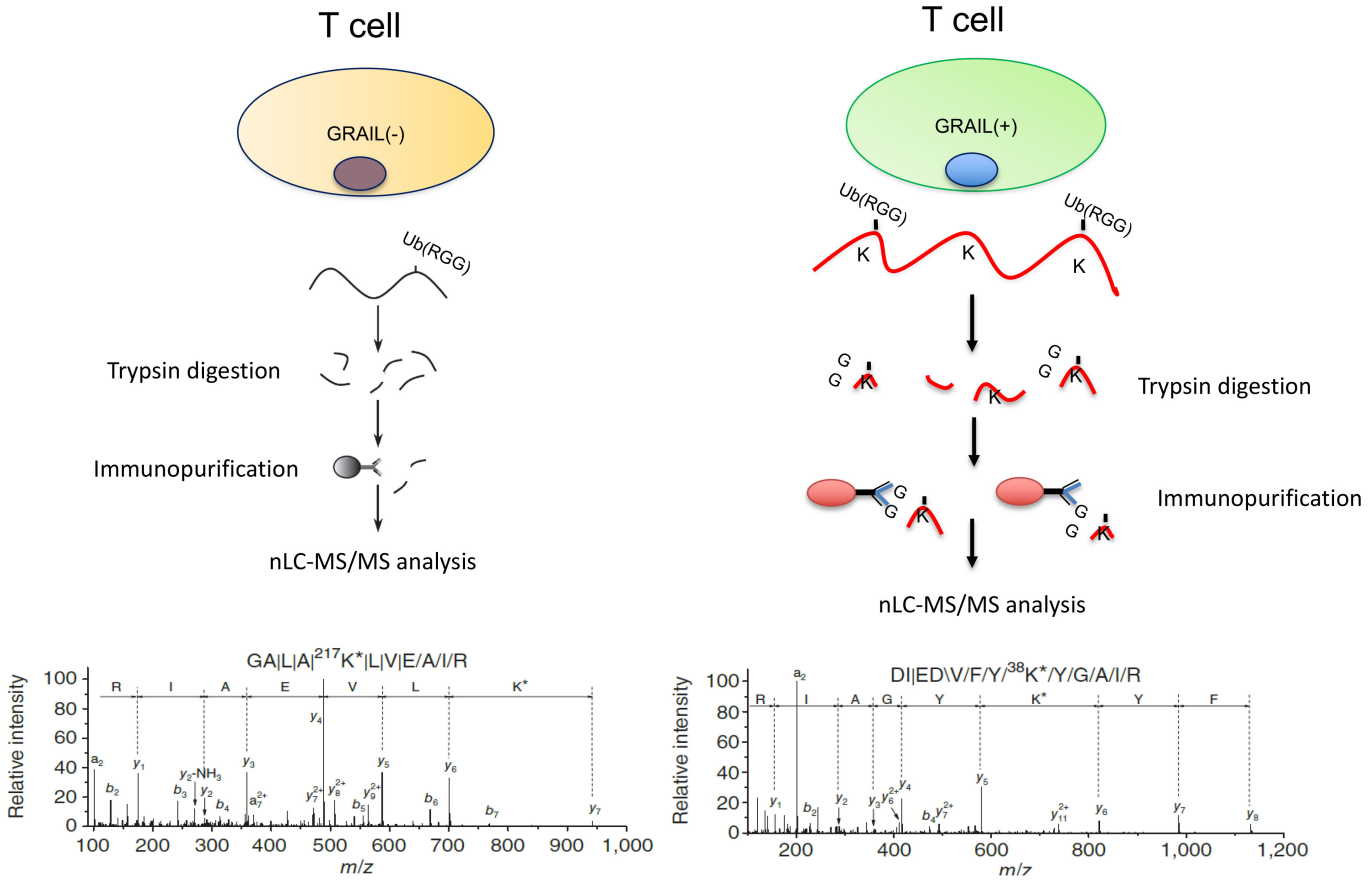
Proteins ubiquitinated by GRAIL. We identified proteins ubiquitinated by GRAIL using a new E3 target identification system from Cell Signaling Technologies. A monoclonal antibody recognizes the di-glycine tag on ubiquitinated lysines on proteins that can then be identified by differential mass spectrometry and sequencing. GRAIL mono-ubiquitinates lysine 724 on Cul5. This is the exact lysine that must be neddylated to allow the Cul5 ring ligase to function. Thus, GRAIL acts as a competitive inhibitor of neddylation to inhibit IL-2R desensitization in Tregs.

To ask how GRAIL expression might play a role in IL-2R desensitization, we looked for proteins ubiquitinated by GRAIL that were involved in IL-2R signaling. To identify a potentially relevant target of GRAIL mediated ubiquitination, we made use of a technique called “global analysis of lysine ubiquitination by ubiquitin remnant immunoaffinity profiling” (25). This technology utilizes a monoclonal antibody that recognizes a di-glycine (K-ϵ-GG) tag that remains on lysines of cytosolic proteins following their ubiquitination (**Figure 4**). Ubiquitinated proteins were then identified by differential mass spectrometry following affinity purification using this monoclonal antibody. We used this technology to compare trypsin digests of Jurkat cells that expressed an inducible form of GRAIL vs. non-induced control Jurkat cells. Cullin5 (Cul5) was identified as a target of GRAIL-mediated ubiquitination. Importantly, the target of GRAIL ubiquitination on Cul5 was K724, the exact lysine that needs to be neddylated to allow the SOCS3/Cul5 CRL to function (**Figure 1B**). Ubiquitination of K724 by GRAIL acts as a competitive inhibitor of neddylation. This results in inhibition of IL-2R desensitization (prolonged IL-2R signaling through pJAK1), allowing the T_reg_ transcriptome for “function” to be stably transcribed.

The ubiquitin E3 ligase GRAIL is crucial to the maintenance of the T_reg_ phenotype. Diminished GRAIL expression seen in the T_regs_ from SLE patients (**Figure 3**) was accompanied by a loss of inhibition of IL-2R desensitization (**Figure 2**) and defective regulatory function when CD4+CD25- effector and CD4+CD25+ regulatory T cells from the SLE patients in **Figure 3** were studied in autologous co-cultures (effector:regulatory T cells ratio 1:1) in 24-well plates and were either left unstimulated (RPMI), or were activated by means of the combination of plate-bound anti-CD3 antibody (5 µg/ml) and soluble anti-CD28 antibody (2.5 µg/ml) for 48 hours. Following this period, cells were harvested for proliferation assays. Cell proliferation was evaluated by FACS (BD LSRII Fortessa; BD Biosciences) according to the CFSE dilution protocol. SLE % average suppression of autologous Teffs was 35.2%; Healthy donors 78.4%, p=0.002.

The Fathman lab had previously shown that GRAIL is associated with and regulated by two isoforms of the ubiquitin-specific protease Otubain; Otub1 and Otub1 alternative reading frame 1, (Otub1-ARF1) (26). In these studies, they reconstituted bone marrow (BM) cells in lethally irradiated OVA reactive T cell receptor-transgenic mice (DO11.10) with DO11.10 BM cells retrovirally transduced to express one of the two isoforms of Otubain. They showed that following antigenic stimulation, Otub1-expressing cells contained negligible amounts of endogenous GRAIL, proliferated well and produced large amounts of IL-2. In contrast, cells expressing Otub1-ARF1 contained large amounts of endogenous GRAIL, demonstrated diminished functional responses; they proliferated poorly and produced undetectable amounts of IL-2 following antigenic stimulation. Thus, these two proteins have opposing functions in controlling the stability of GRAIL and the resultant phenotype of CD4 T cells.

Additional studies from the Fathman lab (27) demonstrated that *Otub1* translation in CD4 T_effs_ was under the control of mTOR. IL-2R driven AKT activation of mTOR, in response to CD4 T_eff_ stimulation, leads to GRAIL degradation in anti-OVA transgenic DO11.10 CD4 T_eff_ cells. In this study it was demonstrated that GRAIL was expressed in quiescent naive mouse and human CD4 T cells and has a functional role in inhibiting T cell proliferation. Following TCR engagement and CD28 co-stimulation, the resultant expression of IL-2 activates an Akt-pathway to activate mTOR leading to selective mRNA translation. Pre-existing mRNA for *Otub1* is translated, resulting in the degradation of GRAIL in CD4 T_eff_ cells, and the proliferation of these cells. Akt activation of mTOR appears to be a critical component of IL-2R signaling regulating GRAIL expression in CD4 T_effs_. Maintenance of GRAIL expression in T_regs_ is crucial for T_reg_ stability and function GRAIL expression is regulated by the two isoforms of Otubain1 (26). Our unpublished data have demonstrated a marked increase in Otubain1-ARF1 mRNA in Tregs. This catalytically inactive isoform blocks GRAIL degradation by competitive inhibition of Otub1to maintain USP8 activity (26). USP8 is a deubiquitinating enzyme that de-ubiquitinates auto-ubiquitinated GRAIL. In Tregs, GRAIL ubiquitinates lysine 724 on Cul5 and blocks its neddylation. When ubiquitinated, lysine 724 can no longer be neddylated. Ubiquitinated K724 on cullin 5 does not allow the ubiquitin transferase on the Cul5 protein E2 to undergo the 50A shift required to be able to transfer ubiquitin to its target pJAK1 (**Figure 1B**) to maintain pJAK1 expression and thus pSTAT5 transcriptional activity (26) (**Figure 1B**).

## How did we identify a drug strategy?

Our studies demonstrated that GRAIL-mediated ubiquitination of Cul5 serves as a negative regulator of the SOCS3/Cul5 CRL and is used by T_regs_ to inhibit IL-2R desensitization (26). Thus, GRAIL mediated ubiquitination of Cul5 in T_regs_ inhibits IL-2R desensitization and allows prolonged IL-2R signaling and prolonged pSTAT5 expression in response to low-dose IL-2. We reasoned that if this is true, it should be possible to restore T_reg_ function in the “defective” T_regs_ from patients, or experimental animals with autoimmune diseases that have diminished GRAIL expression, by blocking neddylation with a small molecule inhibitor of neddylation. This drug would substitute for GRAIL activity. Several small-molecule drugs are inhibitors of the neddylation activating enzyme [*neddylation activating enzyme inhibitors* (*NAEi*)]. We selected one such NAEi, called MLN4924, for our initial studies (14).

**Figure 5:**
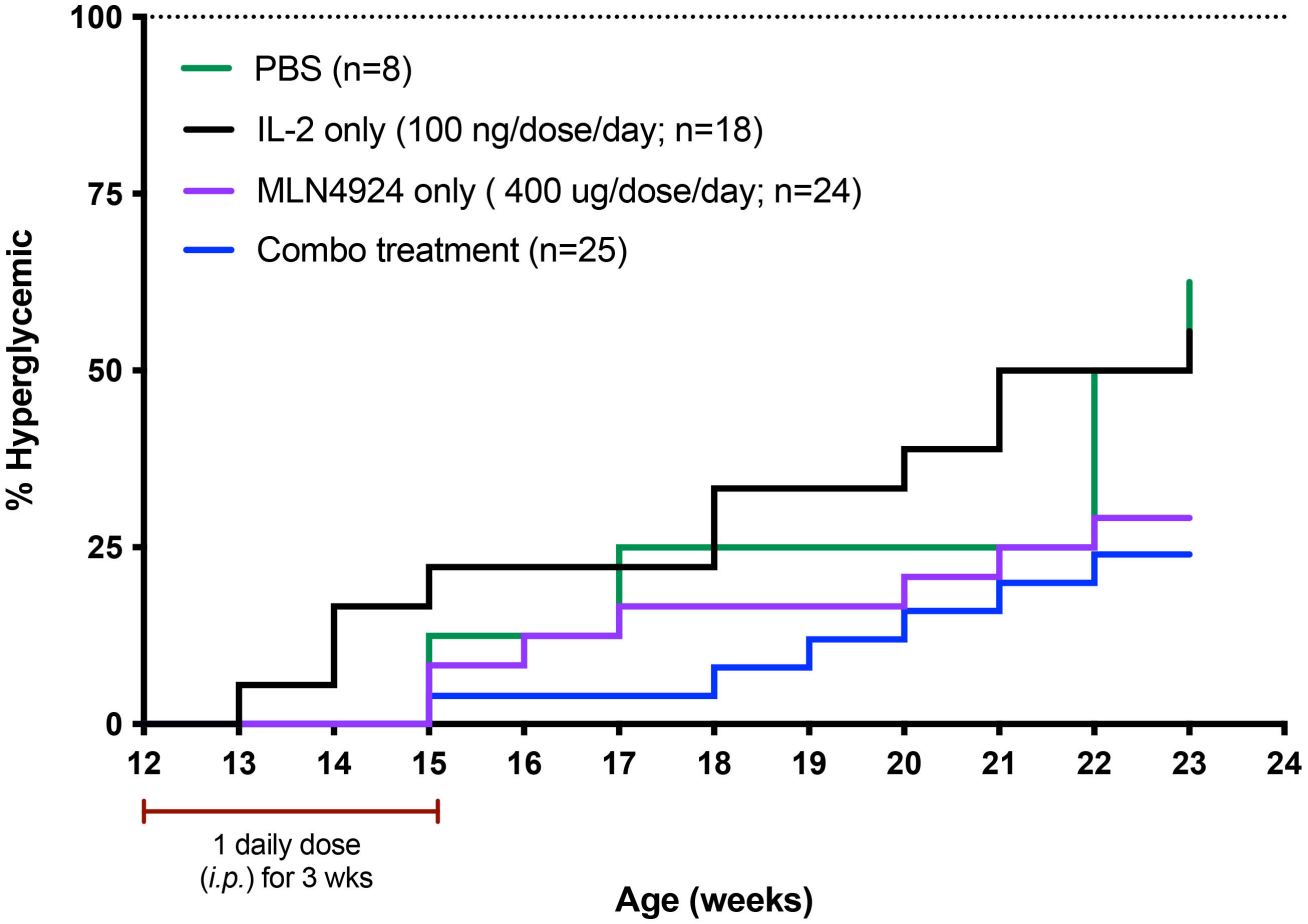
Use of the NAEi MLN4924 to treat NOD disease and undergoes receptor mediated endocytosis. IL-2 binding to the IL-2R triggers cellular pathways leading to second messenger signaling activity. Defective second messenger signal pathways identified in T_regs_ from patients with autoimmune diseases that require inhibition of CRL activity, can be corrected by NAEi’s attached to the PDC by stable linkers that are enzymatically cleaved only inside the target cell to deliver the drug payload to the appropriate cell. B) PDC (ATA-003) consisting of 3 molecules of an NAEi (MLN4924) bound by di-peptide linkers to the thioredoxin portion of the murine IL-2 fusion protein.

There is no previous unifying hypothesis of a common defect leading to the immune dysregulation seen in autoimmune and inflammatory diseases. We reasoned that if diminished GRAIL expression leading to loss of inhibition of IL-2R desensitization was a common defect in mouse models of autoimmunity, where a defect in GRAIL expression had been identified (28), it might be possible to restore T_reg_ function using low-dose IL-2 in combination with an NAEi in these mouse models of disease. Our initial studies in restoring T_reg_ function were in the NOD mouse model of type one diabetes (T1D). T_regs_ from NOD mice had been previously demonstrated to have diminished GRAIL expression (28). We asked if low dose IL-2 in combination with a neddylation activating enzyme inhibitor (NAEi) would have a better effect in preventing disease progression than low dose IL-2 alone. We were able to inhibit progression to hyperglycemia in female NOD mice when we treated them with combination therapy (low-dose IL-2 and the NAEi, MLN) for 21 days, beginning at 12 weeks of age (**Figure 5**). At this age, these mice have pronounced islet inflammation but are normoglycemic. Although the data suggested that the combination was more effective than low-dose IL-2, the systemic administration of the NAEi was toxic at therapeutically effective doses (data not presented).

## Why did we develop a protein drug conjugate approach?

**Figure 6:**
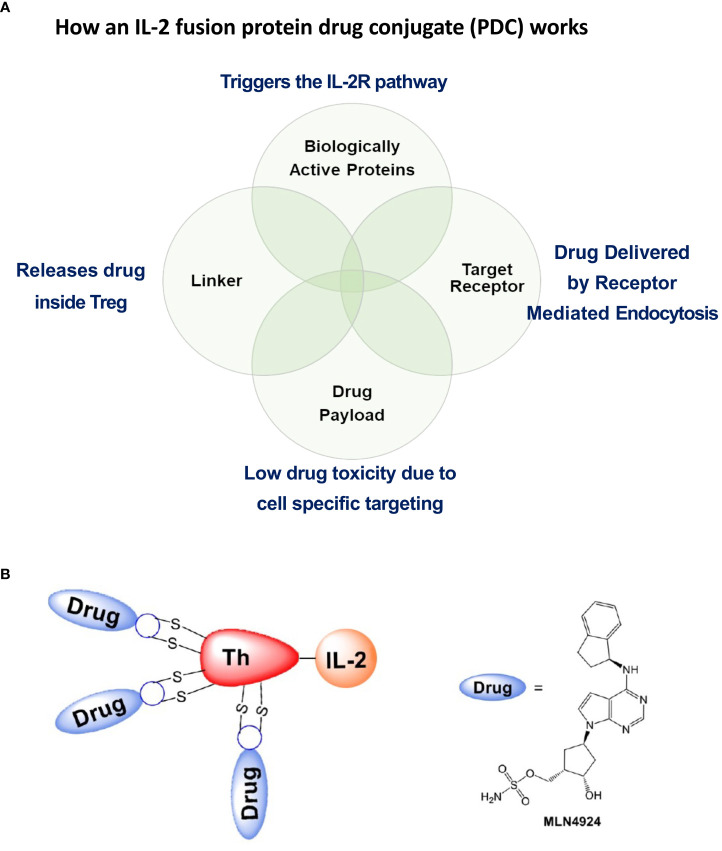
Salient features of the IL-2 fusion PDC. **(A)** The PDC binds to Treg cell surface IL-2R’s that, following PDC binding, undergo receptor mediated endocytosis. IL-2 binding to the IL-2R triggers cellular pathways leading to second messenger signaling activity. Defective second messenger signal pathways identified in Tregs from patients with autoimmune diseases that require inhibition of CRL activity, can be corrected by NAEi’s attached to the PDC by stable linkers that are enzymatically cleaved only inside the target cell to deliver the drug payload to the appropriate cell. **(B)** PDC (ATA-003) consisting of 3 molecules of an NAEi (MLN4924) bound by di-peptide linkers to the thioredoxin portion of the murine IL-2 fusion protein.

In order to decrease the amount of the NAEi given systemically to the mice to restore T_reg_ function, we reasoned that we could develop a protein drug conjugate (PDC) similar to an antibody drug conjugate using the same type of cleavable linker strategy (15) (**Figure 6**). The PDC would specifically target the high affinity IL-2R constitutively expressed on T_regs_ and, following activation-induced receptor mediated endocytosis, deliver the attached drugs directly to signaling endosome of the T_regs_. This is a Trojan Horse model for targeted drug delivery to diminish off target toxicity by using the high affinity receptor (IL-2R) on the target cell (T_reg_) and the ligand (IL-2) to activate the receptor (IL-2R). Once engulfed by receptor mediated endocytosis, the drugs attached by cleavable linkers to the IL-2 fusion protein are specifically released inside the target cell (the T_reg_).

Recombinant IL-2 produced in *E. coli*, was generated by a biotech company, IL-2Rx, by a refolding process from denatured protein recovered from inclusion bodies. To decrease disulfide scrambling during refolding, cysteine 160 in the mouse IL-2 sequence was mutated to serine, leaving only the native disulfide-forming cysteine pair at positions 68 and 115. As documented by antibody-drug conjugate technology development, cysteine-based conjugation vs. lysine conjugation is preferred, due to the randomness of the geographic reactivity of amino-reactive moieties in proteins that contain large numbers of lysines. To increase drug-conjugation potential, IL-2Rx developed a fusion protein consisting of murine IL-2 and murine thioredoxin, with a small, four-amino-acid spacer peptide separating them (**Figure 6B**). This resulted in the production of a largely soluble, small fusion protein, and, after re-bridging the two cysteines on IL-2, allowed three sites on the thioredoxin portion of the PDC for cysteine-based drug conjugation. Using conventional dipeptide linkage, IL-2Rx produced a small molecule–linked PDC, called ATA-003, consisting of an NAEi (MLN4924) with an optimized dipeptide linker and a maleimide moiety attached to the murine IL-2 fusion protein (**Figure 6B**). Analytical methods showed that the fusion protein had three molecules of MLN linked to cysteines by dipeptide cathepsin cleavable bonds to the fusion protein. After passing tests *in vitro* to quality assess the compound, we used ATA-003 to treat animal models of autoimmunity.

## Treatment of NOD mice with the PDC to block progression to hyperglycemia

**Figure 7:**
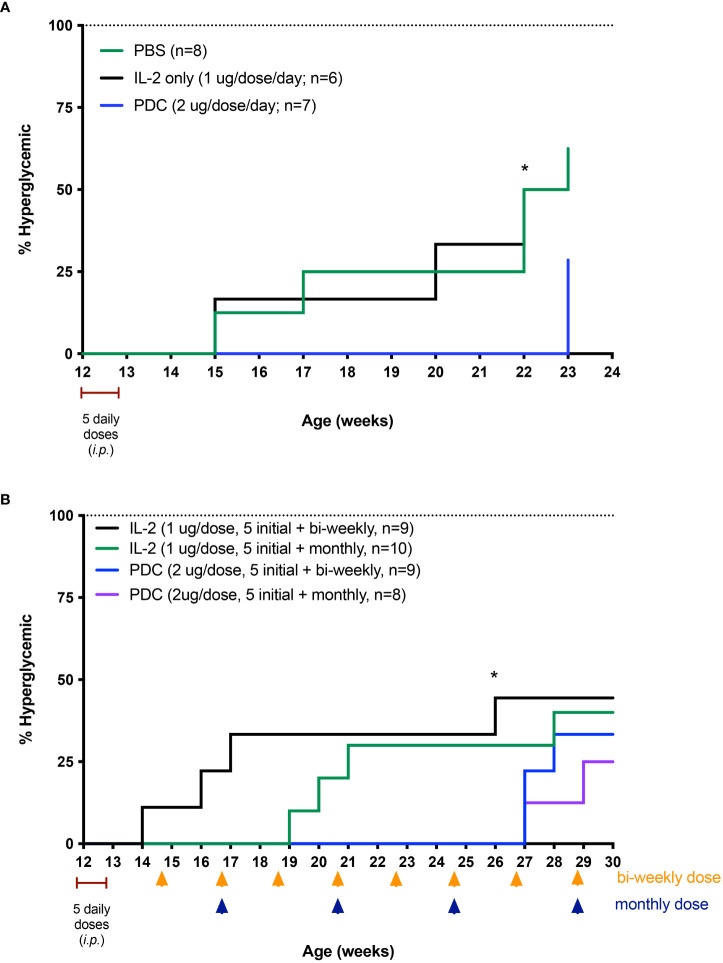
Therapeutic effect of the PDC, protein drug conjugate (ATA-003) in preventing disease in the NOD mouse model of T1D. **(A)** NOD mice were treated *i.p*. with low-dose IL-2 (1 μg; 25,000 IU/day), PDC (2 μg/day), or an equal volume of PBS, phosphate buffered saline (0.1 ml) for 5 consecutive days starting at 12 weeks of age. Disease incidence was significantly different between PDC vs. PBS-treated groups (P < 0.04), and between PDC vs. IL-2 treated groups (P < 0.04; Log rank Mantel-Cox test) at 22 weeks of age. Separate cohorts of NOD mice were treated similarly but with an additional dose of PDC or IL-2 administered every 2 weeks (yellow arrows) or every month (blue arrows) after the initial 5 daily doses **(B)**. Blood sugar was measured weekly until mice became hyperglycemic. Disease incidence was significantly different between biweekly treated PDC vs. biweekly treated IL-2 groups at 26 weeks of age (* P < 0.03; Log rank Mantel-Cox test).

Although the NOD model is a spontaneous model of an autoimmune disease, the pathophysiology is of immune mediated organ destruction leading to loss of the insulin-secreting beta cells of the islets of Langerhans. Since restoring T_reg_ function (tolerance) will not regenerate islet cells, we chose to try and stop progression to hyperglycemia by treating normoglycemic NOD mice at a time consistent with late-stage islet infiltration with immune cells (insulitis). In this study, we treated 12-week-old female NOD mice with the PDC ATA-003 (2 μg per injection equivalent to 25,000 units of IL-2 as the PDC weighs two times as much as IL-2), low dose IL-2 alone (1 μg or 25,000 units per injection), or saline for 5 days and followed the mice for three months following the 5-day treatment. In a second cohort of animals, we administered one single dose of ATA-003 (2 μg) or IL-2 (1 μg 25.000 IU) every 2 weeks or every month after the initial 5-day dose. We found that the PDC treated mice had a much more pronounced response than the mice treated with low dose IL-2 alone, as the PDC treated mice did not progress to hyperglycemia for over 2 months after therapy started, and the administration of a bi-weekly or monthly booster dose delayed disease onset by an additional 4 weeks (**Figure 7**). The PDC was also much more effective in delaying disease onset and progression than systemic treatment with the combination of IL-2 and MLN4924 (**Figure 5**), and there was no detectable toxicity.

## Treatment of (NZB x NZW) F1 lupus nephritis with the PDC

**Figure 8:**
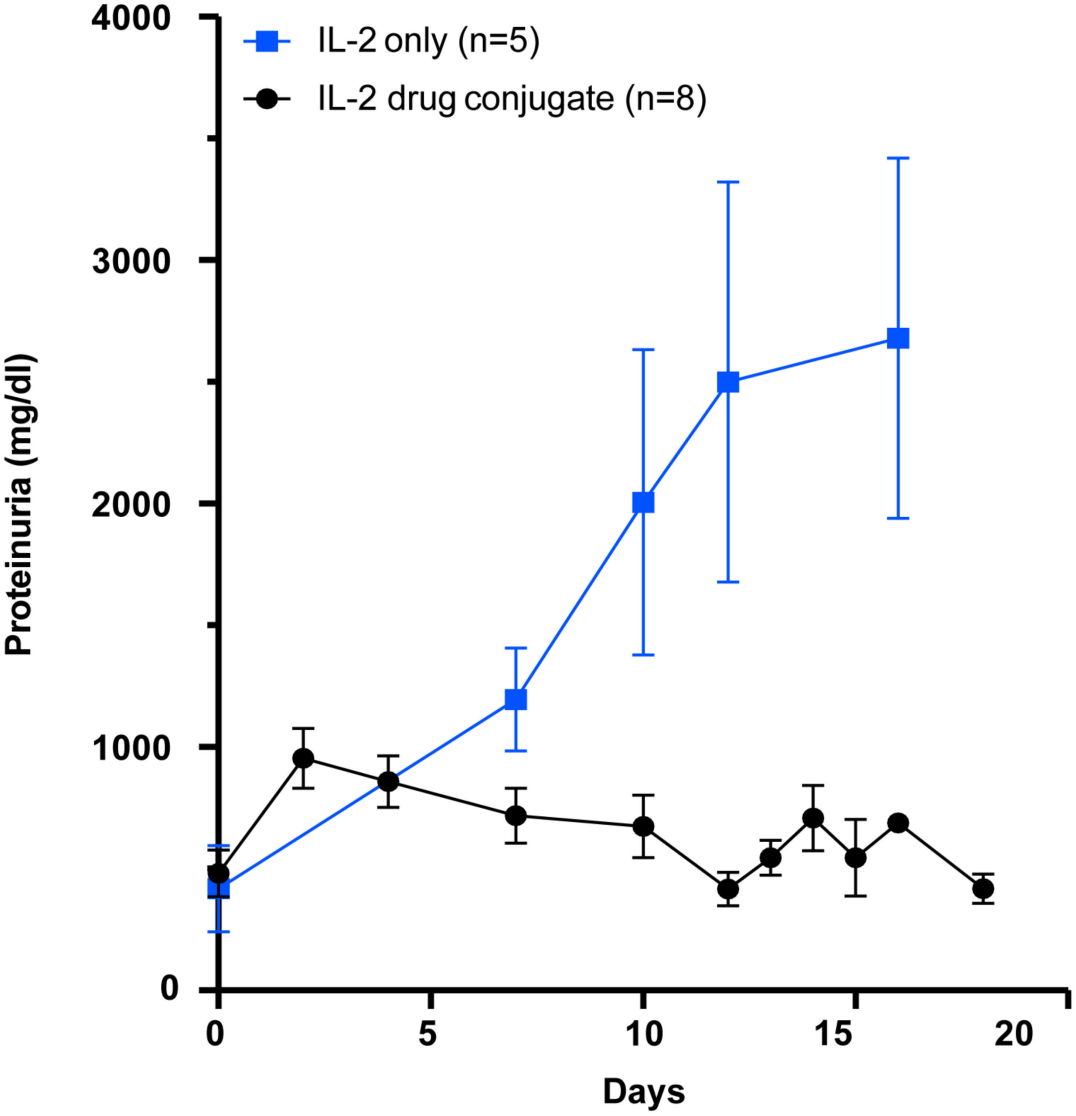
Therapeutic effect of the PDC (ATA-003) in preventing nephritis in an animal model of SLE. Groups of female (NZB × NZW) F1 mice aged more than 24 weeks with a proteinuria score of greater than 500 mg/dl were either given the PDC ATA-003 (2 μg/day; n = 8) or a molar equivalent of low-dose IL-2 (1 μg; 25,000 IU/day; n = 5) for 5 days and followed for 3 weeks. Mice that received saline rapidly progressed to severe proteinuria in a manner similar to the low dose IL-2 treated mice (data not included).

We next asked if our PDC would be better than low dose IL-2 in arresting progression of proteinuria in the (NZB x NZW) F1 (BWF1) mouse model of lupus nephritis. In this study, we asked if our novel PDC could prevent the progression of proteinuria in BWF1 female mice that already had significant kidney disease. Three cohorts of BWF1 female mice were initially studied. At an age between 20 and 24 weeks, the mice developed increasing levels of proteinuria. Once a mouse developed 500 mg/dl of proteinuria, they were randomly assigned to receive either the PDC (2 μg per day), low-dose IL-2 (1 μg per day 25,000 IU), or saline as control, by intraperitoneal injections. The amount of IL-2 in the PDC is the same as in the low-dose IL-2, as the PDC weighs about twice as much as IL-2 alone. The mice were given five, daily *i.p.* injections of the PDC, IL-2, or saline, starting when they had achieved 500 mg/dl proteinuria. Our data demonstrate that the PDC was far superior in blocking progression in proteinuria than the low-dose IL-2 alone (**Figure 8**). Saline treated controls rapidly reached 3 gm/dl and were sacrificed (data not shown).

## Using the PDC to treat a mouse model of asthma

**Figure 9:**
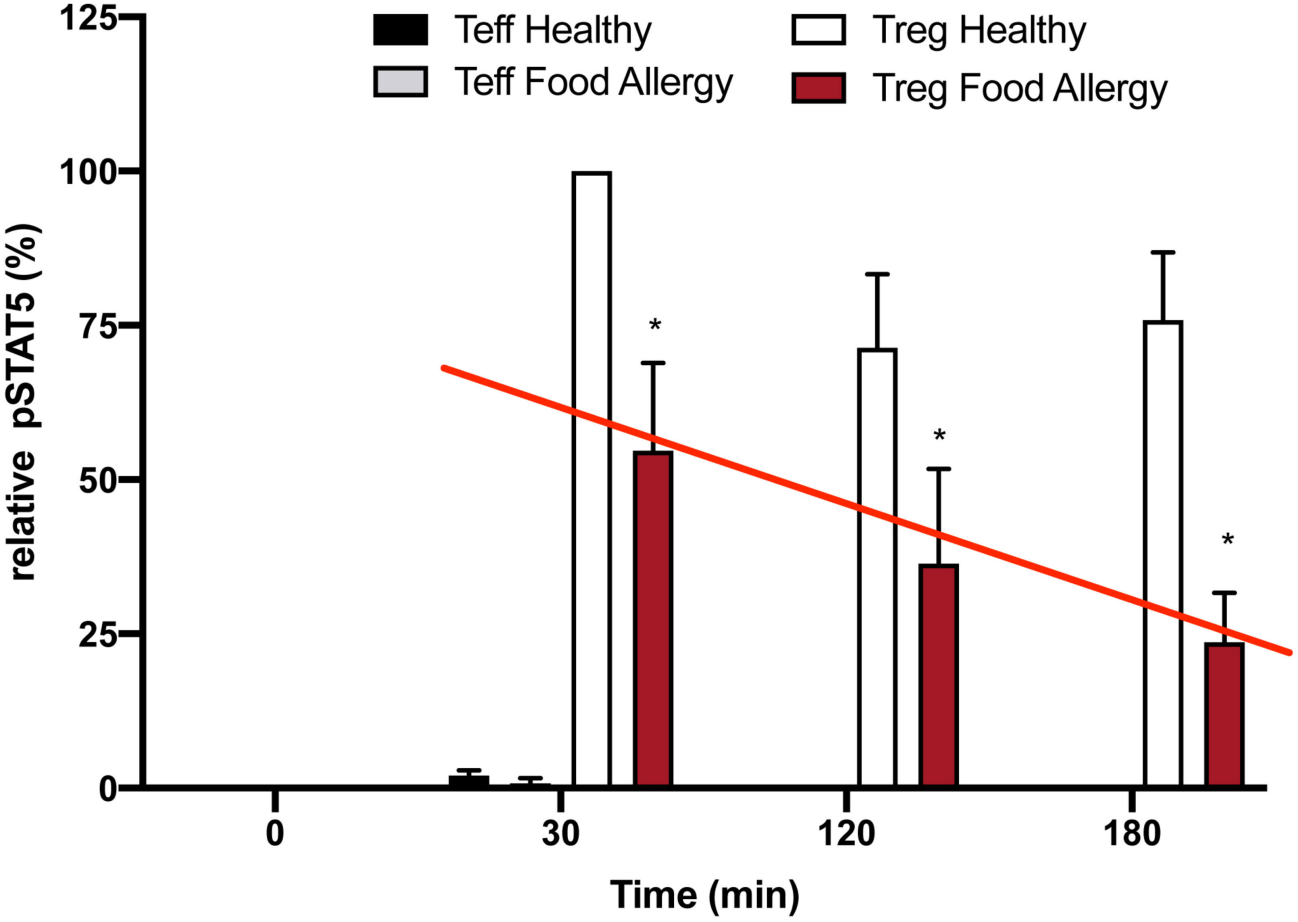
Loss of inhibition of T_reg_ IL-2R desensitization in food allergy patients. Expression of pSTAT5 in human T_effs_ vs. human T_regs_ in healthy and food allergy participants (n=3/group). Expression of pSTAT5 is shown relative to levels measured in T_regs_ of healthy controls stimulated with low dose IL-2 (1 ng; 25 IU/ml) for 30 mins. Cultures were harvested at the indicated times after stimulation and pSTAT5 levels were measured by immunoblotting. Data are normalized to levels measured in 30 min IL-2 stimulated control T_regs._ (mean ± S.D.; p < 0.05 for * with two-way ANOVA).

There is strong evidence in animals that Tregs act as key regulators of allergic diseases. One example is that the adoptive transfer of ovalbumin (OVA) peptide-specific Tregs into the OVA-sensitized mice attenuated airway hyper-responsiveness along with reduced number of eosinophils and production of Th2 cytokines in the lung following allergen challenge (29). A recent review suggested that Tregs are a viable target to attenuate allergic inflammation in asthma (30). It has already been demonstrated that patients with severe food allergy have a defect in T_reg_ function (31). Additionally, low dose IL-2 has been used to treat experimental food allergy (32). We initially asked if T_regs_ from patients with severe food allergy had the same defect in inhibition of IL-2R desensitization as was seen in the T_regs_ from patients with SLE (**Figure 2**). Using Tregs isolated from PBMC’s from patients with severe food allergy, Jennifer Jenks from Kari Nadeau’s lab was able to demonstrate a similar defect in inhibition of the T_reg_ IL-2R response to activation by low dose IL-2 (**Figure 9**). These data again demonstrate that the defect in allergic asthma does not seem to be due to the number of Tregs, but rather to a defect in their function; ineffective second messenger signaling following IL-2R activation.

**Figure 10:**
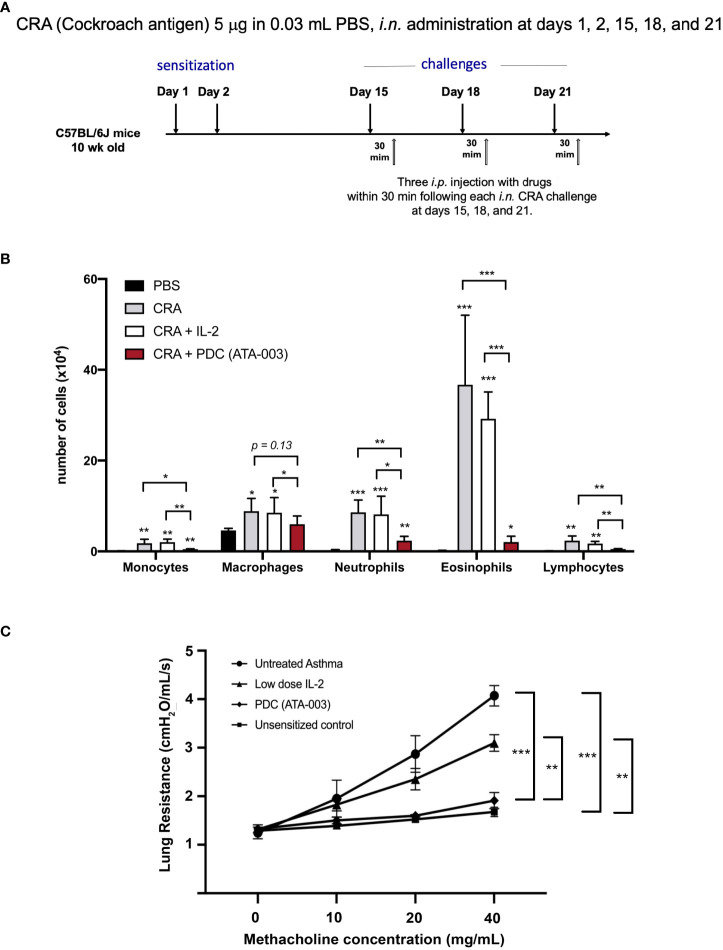
Therapeutic effect to the PDC in a CRA-induced animal model of asthma. **(A)** Asthma was induced in C57/BL6J mice. **(B)** In one cohort of mice, the recruitment of leukocytes into the lung air spaces (diapedesis) was measured 24 h after the last treatment with PDC (2 μg), IL-2 (1 μg 25,000 IU) or an equal volume of saline (0.1 ml). *p < 0.05, ** p < 0.01, *** p < 0.001 compared to PBS -treated group or groups indicated. **(C)** In a second cohort of mice, pulmonary function was evaluated. Lung resistance for each group was compared using two-way ANOVA (Tukey’s multiple comparisons test; ***p < 0.001, **p < 0.01).

To address the potential of the PDC to block an allergen induced allergic response in an animal model, we turned to Mang Yu in Steve Galli’s lab to study the effect of the PDC compared to low dose IL-2 in a mouse model of cockroach antigen (CRA) induced asthma (**Figure 10A**). In the initial study, 1 cohort (n = 5) of 10-week-old female C57BL/l6J (B6) mice with diminished GRAIL expression in their T_regs_ (28), was left untreated as a control for the studies on drug effect. Three cohorts (n = 5/group) were sensitized to the CRA by intranasal administration of 5 μg of CRA in 0.03 mL PBS at days 1 and 2. They were then challenged with the same dose of CRA intranasally at days 15, 18 and 21. Thirty minutes following each of the three challenges, the 3 cohorts of mice received either 0.1 mL of saline *i.p.*, or 0.1 mL of saline containing 1 μg (25,000 IU) of IL-2 *i.p*., or 0.1 mL of saline containing 2 μg of the PDC *i.p*. The mice were then sacrificed 24 hours following the last antigen challenge and treatment, and bronchoalveolar lavage fluid was collected for evaluating the recruitment of leukocytes into the lung air spaces (diapedesis). The results of the study are presented in **Figure 10B**. Remarkably, the number of leukocytes in the alveoli of PDC treated mice was almost the same as the control unsensitized mice when the various leukocyte subsets were examined compared to the extensive diapedesis into the lungs of the saline treated mice. The low dose IL-2 treated mice had a modest decrease in leukocyte recruitment compared to that seen in the PDC treated mice.

A separate cohort of mice (same strain, gender, and age) sensitized, challenged, and treated in an identical manner as above, were assessed for their pulmonary function (**Figure 10C**) using the following methodology: Invasive measurements of airway reactivity in anesthetized, tracheostomized, mechanically ventilated mice were performed 24 hours after the last intranasal exposure (CRA or PBS) by using the BUXCO FivePoint Mouse RC System (Data Sciences International, New Brighton, NM.). Aerosolized, methacholine was administered in increasing concentrations (0, 10, 20, and 40 mg/mL), with individual doses separated by 2 minutes. Lung resistance (R_L_) was continuously computed by fitting flow, volume, and pressure to an equation of motion for each aerosol challenge period, which consisted of a 0.5-minute aerosol exposure and a 1.5-minute period after exposure, as described previously (28).

Tregs have been identified as a good candidate for therapy in allergic asthma. Current studies have used two approaches to attempt to increase Treg numbers in a variety of inflammatory disorders, either using low dose IL-2 or by adoptive transfer of autologous Tregs expanded *ex vivo* back into patients (33). To date there are no convincing studies that the approach of increasing Treg numbers in allergic asthma will work as therapy. Our approach using the PDC described above to restore function to the defective Tregs seen in allergen induced asthma represents a revolutionary form of therapy to treat allergic asthma in man.

## Discussion

Our hypothesis is that the defect in T_regs_ cells seen in autoimmunity and allergic asthma is not due to a deficiency in the number of T_regs_, but rather due to a defect in T_reg_ IL-2R signaling that leads to defective second messenger activity leading to a loss of inhibition of IL-2R desensitization. This defect leads to diminished pSTAT5 and Deptor expression following low dose IL-2 activation of the T_reg_ IL-2R in patients with autoimmune and inflammatory diseases (**Figure 1C**). We hypothesized that this leads to incomplete pSTAT5 transcription of the genes required for T_reg_ function (34). Deptor is an endogenous inhibitor of mTOR activation and is recognized by the F box protein βTrCP of the Cul5/Elongin B complex for polyubiquitination and consequent proteasomal degradation following IL-2 activation of conventional CD25+CD4 T_effs_ (11). mTOR regulates multiple biological processes including those involved in cell growth and proliferation (34). Cell proliferation and differentiation show a remarkable inverse relationship. In conditions where T_reg_ numbers are sufficient, restoration of function in the absence of proliferation may actually be advantageous given the remarkable inverse relationship between cell proliferation and differentiation (35). GRAIL mediated maintenance of Deptor to inhibit mTOR kinase activation in non-autoimmune T_regs_, suggests an important role for this protein in the maintenance of T_reg_ homeostasis (36).

The paradigm of antibody-drug conjugate (ADC) therapy is that delivery of a drug is restricted to the target cell population by means of the specificity of the antibody and the conditional release of the drug warhead in the vicinity of the cell. Our protein-drug conjugate (PDC) model improves on that concept by making use of the high affinity IL-2 receptor in conjunction with low-dose IL-2 activation (**Figure 6**). Our PDC has the advantage of targeting almost exclusively T_regs_ that constitutively express the high affinity IL-2R. Importantly, IL-2R activation by the PDC triggers internalization of the PDC by receptor mediated endocytosis and the drug is delivered to the late endosomes of the T_reg_ where the drug is released by cathepsin cleavage (of the di-peptide bond) to repair the defective signaling pathway activated by IL-2. This can be considered a “Trojan Horse” model for drug delivery. The IL-2 portion of the PDC activates the IL-2R receptor and, following receptor mediated endocytosis (opening the gate of the T_reg_ membrane for targeted drug delivery), delivers the drug directly to the signaling endosome where it corrects the defect in IL-2R signaling. Several publications have now described signaling defects downstream of the IL-2 receptors in T cells from autoimmune subjects both in humans and in animal models (28, 37). This restoration of immune regulation (blockade of disease progression) relies on the fact that a Cullin5-dependent CRL ubiquitin ligase complex is responsible for pJAK1 degradation in the proteasome (35). The Nedd8 activating enzyme inhibitor MLN-4924 blocks the ability of the CRL E2 transferase to deliver ubiquitin to the target of the E3 ligase, pJAK1 (**Figure 1B**). Neddylation of lysine 724 on the cullin backbone of the CRL5 is an absolute requirement for CRL5 function. Neddylation will come to a halt as the inactivated nedd8, bound by the NAEi, accumulates in the targeted T_reg_. In addition to presumably restoring pSTAT5 transcription of the genes required for T_reg_ function, the PDC also blocks Deptor degradation to maintain mTOR in an inactive state and thus stabilizes the T_reg_ phenotype. mTOR activation is required for generation of effector T cells from naïve cells, and its absence or inhibition causes activated naïve T cells to default to a regulatory lineage (38). We are currently evaluating the homologous human fusion protein of the mouse thioredoxin/IL-2 fusion protein PDC as a potential drug candidate to treat several autoimmune diseases and, potentially, allergic asthma. Preliminary studies of isolated activated CD25+ human CD4+T effectors (Teffs) demonstrated that their response to the PDC *in vitro* was similar to their response to a molar equivalent of hIL-2 in proliferation measured by dye dilution 48 hours after stimulation with anti-CD3/28 beads.

## Data availability statement

The original contributions presented in the study are included in the article/supplementary material. Further inquiries can be directed to the corresponding author.

## Ethics statement

The studies involving human participants were reviewed and approved by Stanford School of Medicine. The patients/participants provided their written informed consent to participate in this study. The animal study was reviewed and approved by Stanford School of Medicine.

## Author contributions

CF, LS and LY were the main authors of this article. The text was reviewed by CS, KN, DG-M, JL, JJ and MY and CRH, all of whom participated in deriving the data discussed in the manuscript. All authors contributed to the article and approved the submitted version.
